# Data to support the assessment of the energy efficiency estimation methods on induction motors considering real-time monitoring.

**DOI:** 10.1016/j.dib.2020.105512

**Published:** 2020-04-23

**Authors:** Vladimir Sousa Santos, Juan J. Cabello Eras, Alexis Sagastume Gutiérrez, Mario J. Cabello Ulloa

**Affiliations:** a Energy Department, GIOPEN Research Group, Universidad de la Costa (CUC); b IK4-IKERLAN Technology Research Centre

**Keywords:** Motor efficiency estimation methods, Energy efficiency, Harmonics, Induction motors, Voltage unbalance

## Abstract

The data presented in this article was used to assess and compare the most important methods used to estimate the efficiency during the operation of induction motors at different loads and power supply conditions. The experiment was developed in a test bench including a three-phase induction motor of 1.1 kW (De Lorenzo DL 1021). In addition, an adjustable voltage source, a variable-frequency drive, a resistor, and a magnetic powder brake control unit to regulate the load were used during the experiments. A power quality and energy analyzer (Fluke 435 series 6) was used to measure the electric variables during the experiments. Moreover, for the mechanical measures, the sensors of the brake control unit (De Lorenzo DL 1054TT) and a magnetic powder brake (De Lorenzo DL 1019P) were used. In total, 11 load factors were measured at different operation conditions, including balanced sinusoidal voltage, balanced harmonic voltage, unbalanced sinusoidal voltage and unbalanced harmonic voltage. A total of 10 measures were taken for each load factor at each operation condition. The data presented in this paper can be useful in the development and evaluation of new efficiency estimation methods for induction motors, considering different operation conditions and load factors. Moreover, it can serve to assess the impact of the energy quality on the efficiency of induction motors. The data is related to the manuscript “Assessment of the energy efficiency estimation methods on induction motors considering real-time monitoring” [1].

Specifications table

**Table:** 

Subject	Electrical and Electronic Engineering
Specific subject area	Analysis of energy efficiency in induction motors.
Type of data	Table Figure
How data were acquired	The experiments were developed in a test bench including a three-phase induction motor (De Lorenzo DL 1021), a brake control unit (De Lorenzo DL 1054TT) and a magnetic powder brake (De Lorenzo DL 1019P). The mechanical parameters (i.e. the torque and speed), were measured with the brake control unit and the magnetic powder brake, while the electric parameters were measured with a power quality and energy analyzer (Fluke 435 series 6).
Data format	Raw
Parameters for data collection	Four power supply conditions were considered during the experiments: Balanced sinusoidal voltage. Balanced harmonic voltage. Unbalanced sinusoidal voltage. Unbalanced harmonic voltage. For each condition, the induction motor was operated at 11 load factors, obtained by varying the torque with the magnetic powder brake (De Lorenzo DL 1019P).
Description of data collection	The motor load was increased during the experiments by varying its torque for each of the four power supply conditions defined. The experimental results were measured at the following torque values: 0.50 Nm, 0.75 Nm, 1.00 Nm, 1.25 Nm, 1.50 Nm, 1.75 Nm, 2.00 Nm, 2.25 Nm, 2.50 Nm, 2.75 Nm, and 3.00 Nm. Increasing the torque cause an increment in the temperature of the motor. Thus, before measuring the mechanical and electric parameters it was expected that the temperature stabilized. During the experiments, both the electric and mechanic parameters were measured simultaneously. To guarantee the reliability and consistency of the results, each test was repeated 10 times.
Data source location	Institution: Universidad de la Costa (CUC) City/Town/Region: Barranquilla/Atlántico Country: Colombia
Data accessibility	https://data.mendeley.com/datasets/t4p2wxmm5j/draft?a=8730239d-093f-4cf8-b645-4d2912c7b818
Related research article	Author's name: Vladimir Sousa Santos, Juan José Cabello Eras, Alexis Sagastume Gutierrez, Mario Javier Cabello Ulloa. Title: Assessment of the energy efficiency estimation methods on induction motors considering real-time monitoring. Journal: Measurement DOI: https://doi.org/10.1016/j.measurement.2018.12.080

## Value of the data

• This data is useful to determine the most adequate method to estimate the real-time efficiency of induction motors according to its operating load and power supply characteristics.
• This data can be used by researchers to develop new real-time efficiency estimation methods for induction motors, which consider variable load, harmonics and voltage unbalance.
• This data can be used to further define the influence of harmonics and voltage unbalance on the operation and efficiency of induction motors.

## 1 Data Description

The data correspond to the experiments developed to assess the real-time energy efficiency estimation methods used for induction motors.

Table 1 (see Appendix A) shows the electric and mechanic parameters measured at the 11 load factors for balanced sinusoidal voltage, and the efficiency calculated from the experimental data. Likewise, Table 2 (see Appendix A) shows the electric and mechanic parameters measured for balanced harmonic voltage, and the efficiency calculated from the experimental data. Furthermore, Table 3 (see Appendix A) shows the electric and mechanic parameters measured for unbalanced sinusoidal voltage, and the efficiency calculated from the experimental data. Finally, Table 4 (see Appendix A shows the electric and mechanic parameters measured for unbalanced harmonic voltage, and the efficiency calculated from the experimental data.

Moreover, Table 5 (see Appendix A) shows the power output measured for the induction motor operating under balanced sinusoidal voltage, and the efficiency results estimated with the different methods considered in this experiment. In addition, it includes the estimation error as compared to the efficiency calculated from the experimental values in table 1. Likewise, Table 6 (see Appendix A) shows the power output measured for the induction motor operating under balanced harmonic voltage, and the efficiency results estimated with the different methods considered in this experiment. Additionally, it shows the estimation error as compared to the efficiency calculated from the experimental values in table 2. Similarly, Table 7 (see Appendix A) shows the power output measured for the induction motor operating under unbalanced sinusoidal voltage. Furthermore, it shows the estimation error as compared to the efficiency calculated from the experimental values in table 3. Finally, Table 8 (see Appendix A) shows the power output measured for the induction motor operating under unbalanced harmonic voltage. In addition, it shows the estimation error as compared to the efficiency calculated from the experimental values in table 4.

**Fig. 1:**
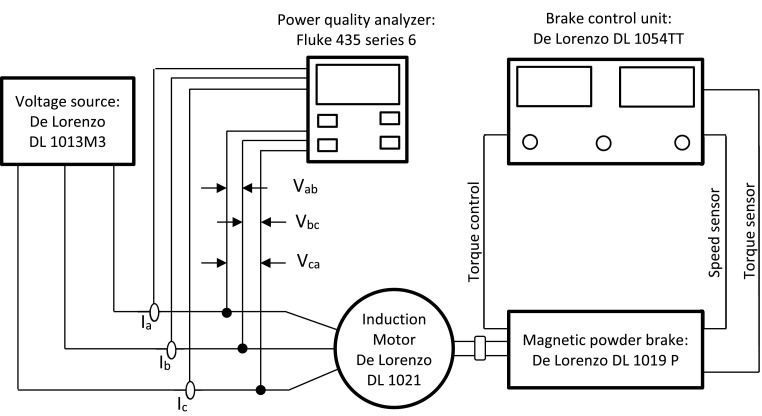
Schematic diagram of the experimental test setup for balanced sinusoidal voltage.

**Fig. 2:**
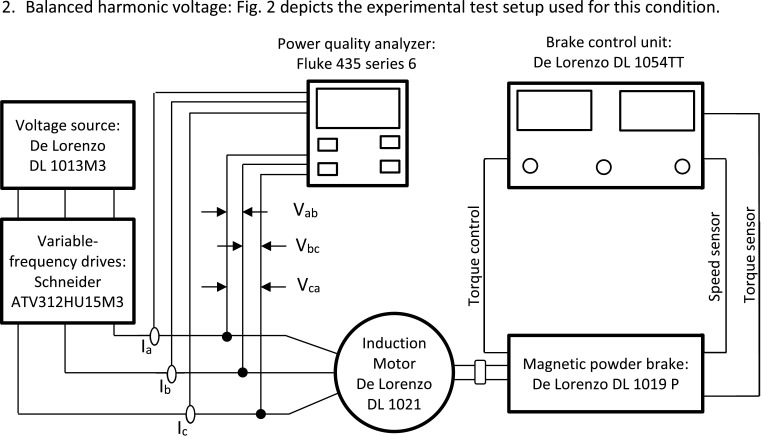
Schematic diagram of the experimental test setup for balanced harmonic voltage condition.

**Fig. 3:**
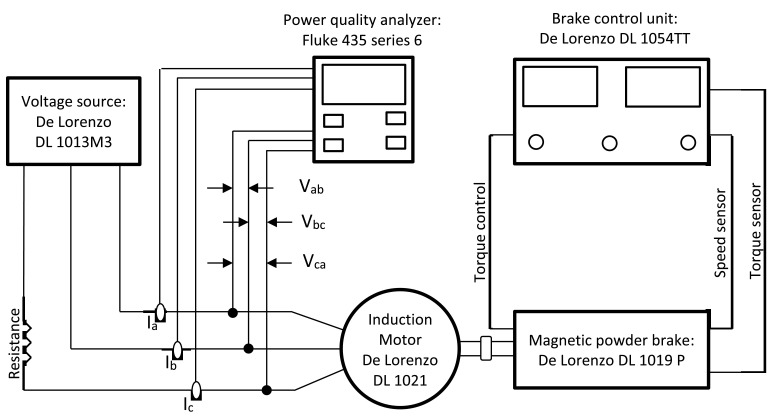
Schematic diagram of the experimental test setup for unbalanced sinusoidal voltage condition.

**Fig. 4:**
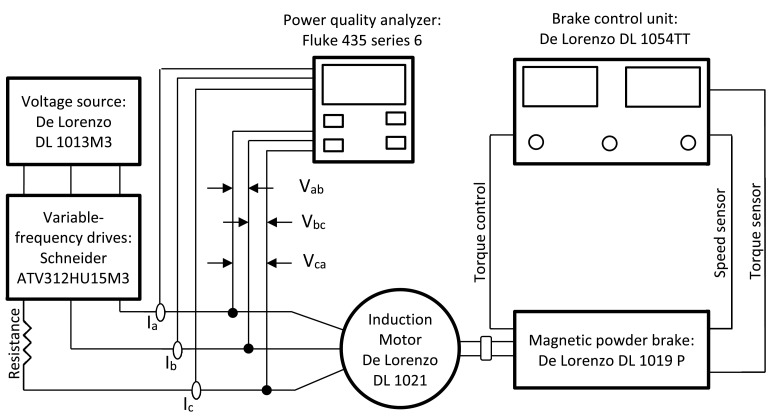
Schematic diagram of the experimental test setup for unbalanced harmonic voltage condition.

**Fig. 5:**
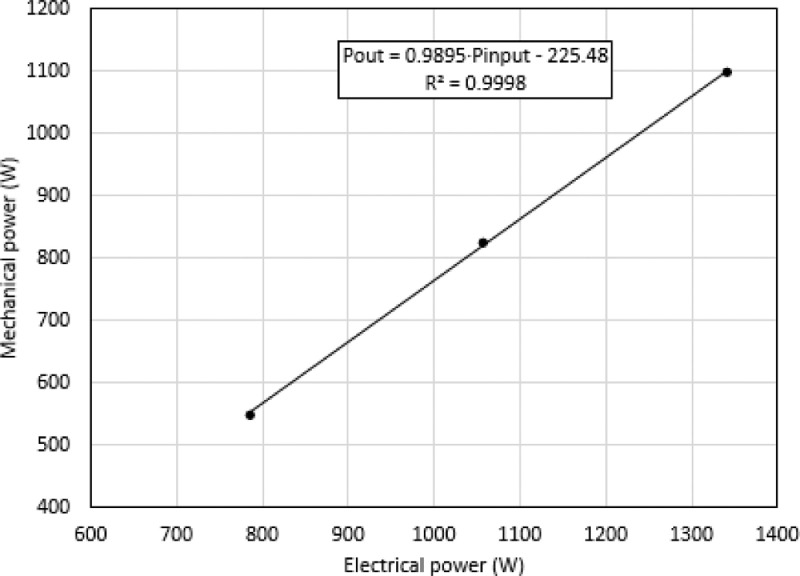
Linear regression of the electric and mechanical power of the De Lorenzo (DL 1021) induction motor.

Table 9 presents the nominal data of the electric motor. Moreover, Fig. 1 shows the experimental test setup used for the balance sinusoidal voltage condition. Fig. 2 shows the experimental test setup used for the balance harmonic voltage condition. Fig. 3 shows the experimental test setup used for the unbalance sinusoidal voltage condition. Fig. 4 shows the experimental test setup used for the unbalance harmonic voltage condition. Fig. 5 shows the linear regression model of electric power vs. mechanical power of the Lorenzo induction motor (DL 1021), obtained from the data in Table 9.

**Table 9:** Nameplate and catalog data of induction motor (De Lorenzo DL 1021)

Parameters	Value
Mechanical power (W)	1,100
Efficiency (%)	82
Voltage (V)	220
Current (A)	3.9
Frequency (Hz)	60
Power factor (p.u)	0.9
Poles	2
Speed (rpm)	3,420
Connection	∆
Insulation class	F
NEMA design	B
Stator resistance r_s_ (at 25°C) (Ω)	4.13
Efficiency (%) Load Factor 100%	82
Efficiency (%) Load Factor 75%	78
Efficiency (%) Load Factor 50%	70
Mechanical power (W) Load Factor 100%	1,100
Mechanical power (W) Load Factor 75%	825
Mechanical power (W) Load Factor 50%	550
Electrical power (W) Load Factor 100%	1,366
Electrical power (W) Load Factor 75%	1,086
Electrical power (W) Load Factor 50%	809

## 2 Experimental Design, Materials, and Methods

The electrical parameters measured during the experiments include:

• Line voltages: V_ab_, V_bc_, V_ca_
• Voltage angles: θ_ab_, θ_bc_, θ_ca_
• Line currents: I_a_, I_b_, I_c_
• Current angles: φ_a_, φ_b_, φ_c_
• Total harmonic distortion voltage (THDV) and individual voltage distortion (V_k_) (apply only for conditions 2 and 4)
• Total harmonic distortion current (THDI) and individual current distortion (I_k_) (apply only for conditions 2 and 4)
• Power factor

A power quality and energy analyzer (Fluke 435 series 6) was used to measure these parameters.

The mechanical parameters included:

• Shaft torque
• Rotor speed

A brake control unit (De Lorenzo DL 1054TT) and a magnetic powder brake (De Lorenzo DL 1019P) were used to measure these parameters.

The electric and mechanic parameters were measured simultaneously.

The mechanical power was calculated with the mechanical torque and speed measured as [2]:

(1) $$P_{\text{out}}=\frac{T_{\text{shaft}}\cdot n_{m}}{9.549}$$

where:

P_out_ – Output or mechanical power (kW)

T_shaft_ – Shaft torque (Nm) n_m_ – Shaft speed (rpm).

The efficiency was calculated with the power measured as [3]:

(2) $$\eta =\frac{P_{\text{out}}}{P_{\text{in}}}\cdot 100\;\left(\%\right)$$

where:

η – Efficiency (%)

P_in_ - Electric power input (kW).

The load factor was calculated as [3]:

(3) $$L_{f}=\frac{P_{\text{out}}}{P_{r}}\cdot 100\;\left(\%\right)$$

where:

L_f_ – Load factor (%)

P_r_ – Rated output power (kW)

For the experiments, an induction motor (De Lorenzo DL 1021) was used. Table 9 shows the nameplate data of the motor.

Four different power supply conditions were considered for the experiments:

1. Balanced sinusoidal voltage: Fig. 1 depicts the experimental test setup used for this condition.

The experimental setup in this case includes a voltage source (De Lorenzo DL 1013M3), which supplies the induction motor (De Lorenzo DL 1021). A magnetic powder brake is used to control the motor load by controlling the torque. The magnetic powder brake is controlled by a brake control unit (De Lorenzo DL 1054TT) that in addition measures the torque and speed. Moreover, the electric parameters are measured with a power quality analyzer (Fluke 435 series 6).

2. Balanced harmonic voltage: Fig. 2 depicts the experimental test setup used for this condition.

In this setup is included a variable-frequency drives, which is connected after the voltage source and operates at the nominal frequency during the experiments.

3. Unbalanced sinusoidal voltage. Fig. 3 depicts the experimental test setup used for this condition.

This setup is like Fig. 1. However, in this case a resistance is connected in series in one of the supply phases.

4. Unbalanced harmonic voltage: Fig. 4 depicts the experimental test setup used for this condition.

This setup includes a variable-frequency drive connected after the voltage source operating at the nominal frequency during the experiments. In addition, a resistance is connected in series to one of the supply phases.

The operation of the induction motor was measured at 11 load factors for each power supply condition.

The load factor was controlled by varying the torque with the magnetic powder brake (De Lorenzo DL 1019 P) between 0.5 and 3.0 Nm at intervals of 0.25 Nm (i.e. 0.50 Nm, 0.75 Nm, 1.00 Nm, 1.25 Nm, 1.50 Nm, 1.75 Nm, 2.00 Nm, 2.25 Nm, 2.50 Nm, 2.75 Nm, and 3.00 Nm). The torque was controlled with the brake control unit (De Lorenzo DL 1054TT). Each experimental test was repeated 10 times.

The efficiency calculated from measured power was compared with the efficiency estimated by applying the nameplate, slip, current and air-gap torque methods.

The application of the nameplate method [4], requires the linear regression of the mechanical power as a function of the electrical power. Fig. 5 shows the linear regression between the mechanical power and the electrical power using the data depicted in Table 9.

The mechanical power output was calculated using the regression model:

(4) $$P_{\text{out}}=0.9895\cdot P_{\text{input}}-225.48$$

To implement the slip method, the mechanical power was calculated as [5]:

(5) $$P_{\text{out}}=P_{r}\cdot \left(\frac{n_{s}-n_{m}}{n_{s}-n_{r}}\right)$$

where: ns - Synchronous speed (rpm) n_m_ – Shaft speed (rpm). n_r_ – Rated shaft speed (rpm).

To implement the current method, the mechanical power was calculated as [6]:

(6) $$P_{\text{out}}=P_{r}\cdot \left(\frac{I_{m}}{I_{n}}\right)$$

where:

I_m_ – Average of the measured current (A)

I_n_ – Nominal current (A).

To implement the air gap torque method, the mechanical power was calculated as [7]:

(7) $$P_{\text{out}}=\frac{2\cdot \pi \cdot T_{\text{ag}}\cdot n_{m}}{60}-\left(P_{\text{fe}}+P_{\text{fw}}\right)-P_{\text{sll}}$$

Where the T_ag_ is calculated as:

(8) $$T_{\text{ag}}=\frac{\sqrt{3}\cdot P}{6}\left\{\left(i_{a}-i_{b}\right)\cdot \int \left[v_{\text{ca}}+r_{s}\cdot \left(2\cdot i_{a}+i_{b}\right)\right]\text{dt}+\left(2\cdot i_{a}+i_{b}\right)\cdot \int \left[v_{\text{ab}}-r_{s}\cdot \left(i_{a}-i_{b}\right)\right]\text{dt}\right\}$$

where

P – Number of poles i_a_ – Instantaneous stator phase current of phase a (A) i_b_ – Instantaneous stator phase current of phase b (A) v_ab_ – Instantaneous stator line voltage of line ab (V) v_ca_ – Instantaneous stator line voltage of line ca (V)

P_fe_ – Core losses (W)

P_fw_ – Friction and windage loss (W)

P_sll_ – Stray load loss (W)

T_ag_ – Air-gap torque (Nm)

The combined no-load losses in the air gap torque method (i.e. P_fe_ + P_fw_) are estimated at 3.5% of rated power output, while P_sll_ is estimated as 1.8% of rated output power [8].

The error between the efficiency calculated with the values of P_out_ measured experimentally, and the efficiencies estimated with the P_out_ calculated with Eqs. 4, 5, 6, and 7 is determined as [9]:

(9) $$\text{Error}=100\cdot \frac{\eta_{e}\;-\;\eta_{m}}{\eta_{m}}$$

where:

ɳ_e_ – Efficiency calculated with a value of P_out_ estimated with equation 4, 5, 6, or 8

ɳ_m_ – Efficiency calculated with the experimental value of P_out_

## Conflict of Interest

The authors declare that they have no known competing financial interests or personal relationships that could have appeared to influence the work reported in this paper.

## Appendix

Appendix Tables are available in the mendeley link: https://data.mendeley.com/datasets/t4p2wxmm5j/draft?a=8730239d-093f-4cf8-b645-4d2912c7b818.

## Appendix Supplementary materials

Image, application 1
